# A rare *PALB2* germline variant causing G2/M cell cycle arrest is associated with isolated myelosarcoma in infancy

**DOI:** 10.1002/mgg3.1746

**Published:** 2021-08-12

**Authors:** Angelina Beer, Ricardo Beck, Anne Schedel, Malte von Bonin, Jörn Meinel, Ulrike Anne Friedrich, Maria Menzel, Meinolf Suttorp, Sebastian Brenner, Guido Fitze, Björn Lange, Ralf Knöfler, Julia Hauer, Franziska Auer

**Affiliations:** ^1^ Neonatology and Pediatric Intensive Care Department of Pediatrics University Hospital Carl Gustav Carus Dresden Germany; ^2^ Department of Pediatric Surgery University Hospital Carl Gustav Carus Dresden Germany; ^3^ Pediatric Oncology and Hematology Department of Pediatrics University Hospital Carl Gustav Carus Dresden Germany; ^4^ Medical Clinic I University Hospital Carl Gustav Carus Dresden Germany; ^5^ German Cancer Consortium (DKTK Dresden Germany; ^6^ German Cancer Research Center (DKFZ Heidelberg Germany; ^7^ Institute of Pathology University Hospital Carl Gustav Carus Dresden Germany; ^8^ Medical Faculty Pediatric Hematology & Oncology Technical University Dresden Germany; ^9^ National Center for Tumor Diseases (NCT) Dresden Germany; ^10^ German Cancer Research Center (DKFZ) Heidelberg Germany

**Keywords:** extramedullary myelogenous leukaemia, myeloid sarcoma, tumour suppressor, PALB2

## Abstract

**Background:**

Isolated myelosarcoma of infancy is a rare presentation of acute myelogenous leukaemia (AML). Because of its rarity and early onset in infancy underlying genetic predisposition is potentially relevant in disease initiation.

**Methods and Results:**

We report an oncologic emergency in an infant with thoracic and intraspinal aleukaemic myeloid sarcoma causing acute myelon compression and lower leg palsy. Whole‐exome sequencing of the patient's germline DNA identified a rare *PALB2* (OMIM 610355) variant (p.A1079S), which is located in a domain critical for the gene's proper function within the homology‐directed repair pathway. In line with potential DNA damage repair defects mediated by the PALB2 deregulation, the patient's fibroblasts showed increased sensitivity towards radiation and DNA intercalating agents.

**Conclusion:**

Therefore, we suggest *PALB2* p.A1079S as a pathogenic variant potentially contributing to the here observed patient phenotype.

## Graphical abstract

We present the finding of an infant with thoracic and intraspinal aleukemic myeloid sarcoma with a rare germline PALB2 variant (p.A1079S). The identified variant is located in a domain critical for the gene's proper function within the homology‐directed repair pathway. A functional role of the variant is further supported by increased sensitivity towards irradiation and DNA intercalating agents observed in primary cells from the patient carrying PALB2 p.A1079S.

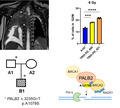

## 1 INTRODUCTION

PALB2 is a tumour suppressor that plays a critical role in homologous recombination repair (HR). Mechanistically, it mediates the recruitment of BRCA2 and RAD51 to sites of DNA damage and functions downstream of BRCA1 (Hanenberg & Andreassen, 2018; Xia et al., 2006). Apart from HR, PALB2 regulates S and G2 DNA damage checkpoints and suppresses genome instability by protecting transcriptionally active genes from genotoxic stress (Hanenberg & Andreassen, 2018; Menzel et al., 2011).

Bi‐allelic inactivating germline mutations in PALB2 that at least partly truncate the BRCA2‐binding C‐terminal WD40 domain, result in the Fanconi anaemia (FA) subtype N. These patients display a severe FA phenotype with a high cancer incidence, including acute myeloid leukaemia (AML) before the age of 5 years (Reid et al., 2007; Xia et al., 2007).

While AML accounts for about 4% of all malignant diseases in childhood, myeloid sarcoma, also called ‘granulocytic sarcoma’ or ‘chloroma’, is even rarer and can occur as an extramedullary myeloid tumour (EML) in up to one‐fifth of paediatric AML. Such tumours can develop in 2–4% of children with AML prior to any bone marrow involvement and are therefore often a diagnostic challenge (Bisschop et al., 2001; Dusenbery et al., 2003; Kaatsch & Spix, 2019; Kobayashi et al., 2007; Pramanik et al., 2018; Reinhardt & Creutzig, 2002; Stove et al., 2017).

Here, we report an infant with an extended atypic localization of myeloid sarcoma as isolated manifestation of an AML with a paternally inherited rare *PALB2* germline variation.

## 2 RESULTS

### 2.1 Case description

**FIGURE 1:**
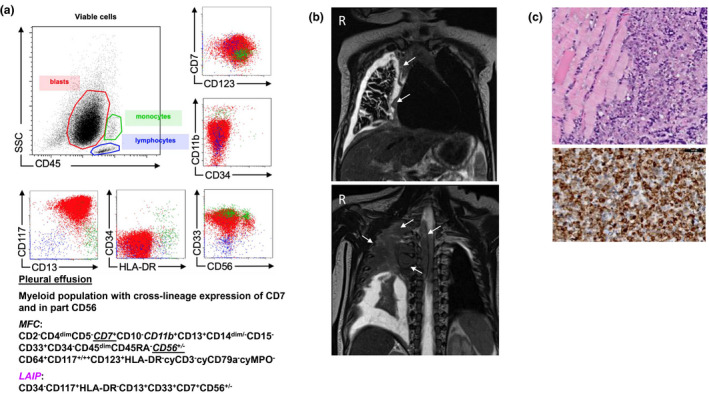
Clinical presentation of isolated myelosarcoma of infancy. (a) Lymphocyte typing of the pleural effusion with a myeloic blast population with aberrant expression of CD7 and in part CD56; MFC = multiparameter flow cytometry, LAIP = leukaemia‐associated immunophenotype. (b) Initial MRI of the chest, coronary axis, at the level of the right ventricle (top) and spinal canal: extensive malignant tumour alongside the right‐sided pleura with partial infiltration of the thoracic wall, infiltration of the mediastinum and per continuity through the neuroforamina into the extra‐axial intraspinal compartment and alongside the big vessels to the intra‐abdominal compartment. The widespread intraspinal portion of the tumour translocated and compressed the myelon. Tumour mass is marked by white arrows (c) Histopathological features of the transthoracic tumour biopsy, HE, 20x: Infiltration of the interthoracic muscles by a small, blue and round‐cell neoplasia; Immunophenotypic pattern of the thoracocentesis, cell block. CD 117, 20x; numerous medium‐sized cells with pleomorphic nuclei, prominent nucleoli and plenty apoptosis figures

A 7‐month old male infant was transferred to our clinic in deteriorating clinical condition over the last 2 days, with massive tachydyspnea. The physical examination on admission was remarkable for a silent chest, reduced percussion sound on the right side and a very mild partial paralysis of both lower limbs, while laboratory results were unremarkable (Table S1). The ultrasound and chest x‐ray revealed a large right‐sided pleural effusion (Figure S1) with monoblastic cells, aberrant expression of CD7 and in part CD56 (Figure 1a). Consequently, we performed a CT (Figure S2) and MRI of the chest (Figure 1b), which showed a tumour involving the right pleura with infiltration of the thoracic wall, the mediastinum and further ranging through the neural foramina to extra‐axial intraspinal and growing intra‐abdominal along the blood vessels. The intraspinal proportion led to a compression of the myelon causing partial palsy of the legs.

Bone marrow morphology revealed normal erythropoiesis, myelopoiesis and megakaryopoiesis with 7.5% of myelomonocytic blasts. No increased proportion of myeloid associated blasts (0.6%) was identified by flow cytometry. Cytogenetics verified a chromosome 6q deletion. The transthoracic tumour biopsy revealed a tumour cell population with positivity for CD117, partially for CD14 and focally for CD45. The Ki67 proliferation index was 80% (Figure 1c). In synopsis with the radiological, laboratory, flow cytometric and histopathological findings, we finally diagnosed an isolated extramedullary manifestation of a myelomonocytic sarcoma. The family history was unremarkable for neoplasia except for a great‐grandfather with skin cancer.

The patient was initially stratified into the intermediate risk group of the treatment protocol AML‐BFM 2012 and therapy was started in an emergency because of myelon compression. The palsy of the lower limbs declined during the pre‐phase with cytarabine and MRI confirmed the rapid regression of the spinal cord compression. After the first induction cycle, 16% blasts were detectable in the bone marrow cytology, therefore the patient was re‐stratified into the high‐risk group with an indication for allogeneic stem cell transplantation (SCT) in the first complete remission. After the fourth cycle, he underwent SCT and received PBSCs from a matched unrelated donor. As an early complication, he developed acute Graft‐versus‐Host‐Disease of the skin grade I on day +9 and an early CMV reactivation on day +12. He was discharged on day +40, the donor chimerism on day +100 was 100% and following MRIs did not show any residual signs of the EML now 17 months post‐SCT.

### 2.2 Germline sequencing identifies a rare *PALB2* variant (p.A1079S)

**FIGURE 2:**
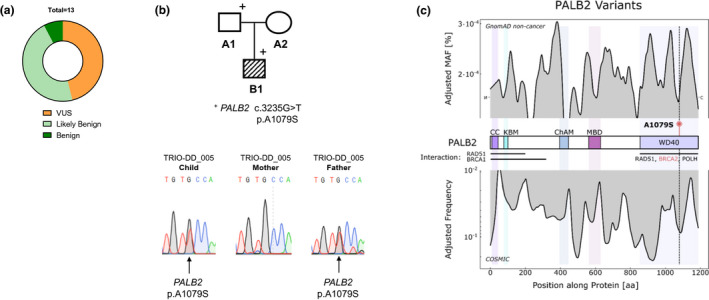
A rare paternally inherited *PALB2* p.A1079S germline variant. (a) Obtained variants classified by CPSR; variants of unknown significance (VUS) n = 6, likely benign n = 6, benign n = 1. (b) Pedigree of analysed family with the identified heterozygous *PALB2* variant and Sanger validation of *PALB2* p.A1079S within the family. (c) Distribution of mutational frequencies along *PALB2*. Upper graph: combined minor allele frequencies of all *PALB2*‐coding germline variants from the gnomAD non‐cancer database represented as LOWESS fit. Lower graph: combined and smoothed (LOWESS) occurrences of *PALB2* somatic tumour mutations from the COSMIC database. The location of the *PALB2* p.A1079S germline variant within the WD40 repeat region of the gene is indicated as a red lollipop, showing a low occurrence within the healthy population, while the position is found to be frequently mutated in cancer samples

Despite a neoplasia unremarkable family history, the combination of a rare presentation of AML and early onset in infancy points towards a potential germline predisposition as an initiator of the disease. Therefore, TRIO sequencing, encompassing whole‐exome sequencing of the patient as well as his parents, was carried out. By applying the CPSR engine (Nakken et al., 2019), no proven pathogenic or likely pathogenic variants were found (Figure 2a). Nevertheless, a variety of possibly damaging variants of unknown significance (VUS) were identified (Table S2). Taking minor allele frequency (MAF) as well as potential functional consequences into account, the heterozygous germline variant *PALB2* c.3235G>T, which was validated in the boy as well as his father (Figure 2b), was chosen for further analysis. While the missense variant is extremely rare (MAF < 0.01) it leads to exchange from alanine to serine in Exon 12 (p.A1079S), which is located within the fifth WD40‐repeats region of the protein. This domain was shown to interact with RAD51, BRCA2 and POLH and is required for POLH DNA synthesis stimulation (Buisson et al., 2014), while the mutation is predicted to be damaging with a CADD score of 22.9. To assess the pathogenicity of the variant further, we correlated mutational frequencies of each *PALB2* nucleotide by comparing its germline variation in healthy individuals (Gnom‐AD v2.1.1 non‐cancer *n* = 118.479) and somatic mutations in cancer (COSMIC v91 *n* = 37,221). As expected, *PALB2* c.3235G>T and its surrounding nucleotides are rarely mutated in the germline of healthy individuals, while they show a high frequency within cancer (Figure 2c).

### 2.3 *PALB2* p.A1079S fibroblasts show increased sensitivity towards DNA damage

**FIGURE 3:**
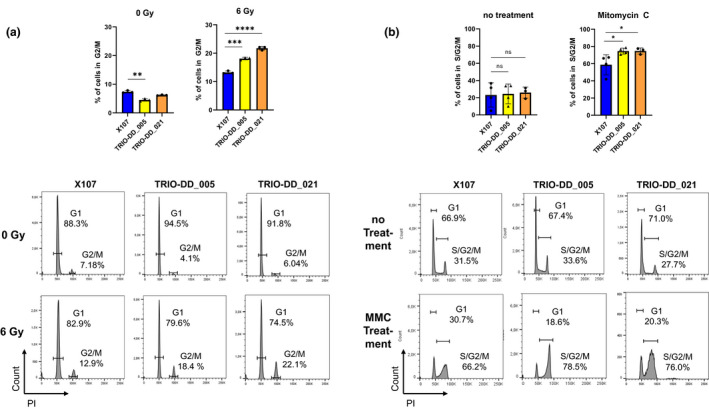
Cells carrying *PALB2* p.A1079S show increased sensitivity towards irradiation and mytomycin C. (a) Upper: irradiation of patient (TRIO‐DD_005), negative control (X107) and positive control (TRIO‐DD_021; BRCA p.Met1?) fibroblasts with 6 Gy and subsequent cell cycle analysis after 48 h. Fibroblasts from the patient respond with a significant increased G2/M arrest compared to the negative control. Plotted is the mean with SD percentage of cells in G2/M phase of three independent biological replicates. Lower: representative flow cytometry histograms of the cell cycle analysis, showing an increased G2/M arrest in TRIO‐DD_005 and TRIO‐DD_021 fibroblasts after irradiation compared to X107. (b) Mitomycin C (MMC) treatment of patient and control fibroblasts with 0.1 mg/ml for 5 min and subsequent cell cycle analysis after 24 h. Likewise the positive control, fibroblasts from the patient responded with a significantly increased S/G2/M arrest compared to X107. Plotted is the mean with SD percentage of cells in S/G2/M phase of independent biological replicates (n = 4TRIO‐DD_005 and X107, n = 3TRIO‐DD_021). Lower: representative flow cytometry histograms of the cell cycle analysis showing an increased S/G2/M arrest in TRIO‐DD_005 and TRIO‐DD_021 fibroblasts after MMC treatment compared to X107. P‐values are indicated with asterisks and were calculated using Student's *t* test (ns = not significant; *p ≤ .05; **p ≤ .01; ***p ≤ .001; ****p ≤ .0001)

It has previously been shown that due to its essential function in HR, PALB2 loss confers hypersensitivity to ionizing radiation and to DNA interstrand crosslinking agents like Mitomycin C (Park et al., 2014; Xia et al., 2006). To assess whether *PALB2* p.A1079S hampers the cells’ capacity for DNA double‐strand break‐induced HR, we subjected primary fibroblasts of the patient, of healthy control and a patient with a known heterozygous pathogenic *BRCA2* mutation (p.Met1?; rs80358650) as a positive control to 6 Gy of ionizing irradiation. After 48 h, the cells’ response was measured using cell cycle analysis. Consistent with a functional effect of the identified *PALB2* variant, fibroblasts carrying p.A1079S showed a significantly higher percentage of cells arrested in G2/M phase compared to the healthy control (Figure 3a). Likewise, Mitomycin C treatment showed an increased sensitivity in cells harbouring the identified *PALB2* variant compared to the healthy control, with patient cells being arrested at S/G2/M to similar levels like fibroblasts carrying the pathogenic *BRCA2* start lost mutation (Figure 3b).

## 3 DISCUSSION

The diagnosis of a primarily extramedullary manifestation of an AML can be very complex and, dependent on the localization of the myeloid sarcoma, can even lead to an oncological emergency. To our knowledge, this case is the first description of an infant with a vast isolated extramedullary medio‐thoraco‐spinal tumour infiltration as the first manifestation of an AML. The rarity of presentation and the early onset suggest an underlying genetic predisposition.

Carriers of heterozygous germline variants within the HR pathway have an increased lifetime risk to develop breast, ovarian and other cancers (Antoniou et al., 2014; Roy et al., 2011). The identified *PALB2* p.A1079S variant is located within a highly relevant functional domain, in close proximity to the breast cancer‐associated functional missense mutations p.L939W, p.T1030I and p.L1143P and likewise shows increased cellular sensitivity to ionizing radiation (Park et al., 2014). Moreover, the identified variant was previously reported in a breast cancer patient (Damiola et al., 2015), further supporting its functional consequence in tumour pathogenesis. Nevertheless, since *PALB2* p.A1079S was inherited from the healthy father, the variant either displays incomplete penetrance or requires additional genetic or environmental stressors to facilitate malignant transformation. In case of additional contributing variants, the likely benign, maternally inherited *POLQ* p.(L1720F) variant (MAF = 0.002; Table S2) could potentially potentiate the effect of deregulated *PALB2*, as *POLQ* is an important part of the microhomology‐mediated end‐joining (MMEJ), a back‐up double‐strand‐break repair mechanism used in cells with a defective HR pathway (Sfeir & Symington, 2015). Likewise, the more commonly found maternally inherited *FANCC* VUS p.(PS26F) (MAF = 0.004; Table S2) could act as a synergistic double hit in the patient.

Taken together, our case explicates the complexities involved in diagnosing a primarily EML and showcases the oncological emergency and complications that can be entailed. The identified *PALB2* variant and its functional consequence in G2/M arrest provide pathogenetic evidence for the early onset and rare presentation of the here described extramedullary AML.

## 4 ETHICS STATEMENT

Families were consented according to the Ethical Vote EK 181042019 and in line with the Declaration of Helsinki.

## 5 AUTHOR CONTRIBUTIONS

Conception and design of the project were prepared by JH and FA; development of methodology were performed by AB, RB, AS, UAF; data were acquired by AB, RB, AS, UAF, MM, FA; management of patient information was performed by AB, RB, MvB, JM, MS, SB, GF, BL, RK, JH; responsible for analysis and interpretation of data (e.g., statistical analysis, biostatistics, computational analysis): AB, RB, AS, UAF, MM; writing, review, and/or revision of the manuscript: AB, RB, AS, MS, JH, FA; administrative, technical or material support (i.e., reporting or organizing data, constructing databases) was compiled by UAF.

## Supporting information

Fig S1‐S3‐Table S1‐S2

Supinfo S2
