# Supplementary material for: A rare PALB2 germline variant causing G2/M cell cycle arrest is associated with isolated myelosarcoma in infancy
Source: Mol Genet Genomic Med. 2021 Aug 12;9(9):e1746. doi: 10.1002/mgg3.1746 (PMC8457705; doi:10.1002/mgg3.1746)
Supplement: Supplementary file 1 — Fig S1‐S3‐Table S1‐S2 [file MGG3-9-e1746-s001.pptx]

## Slide 1
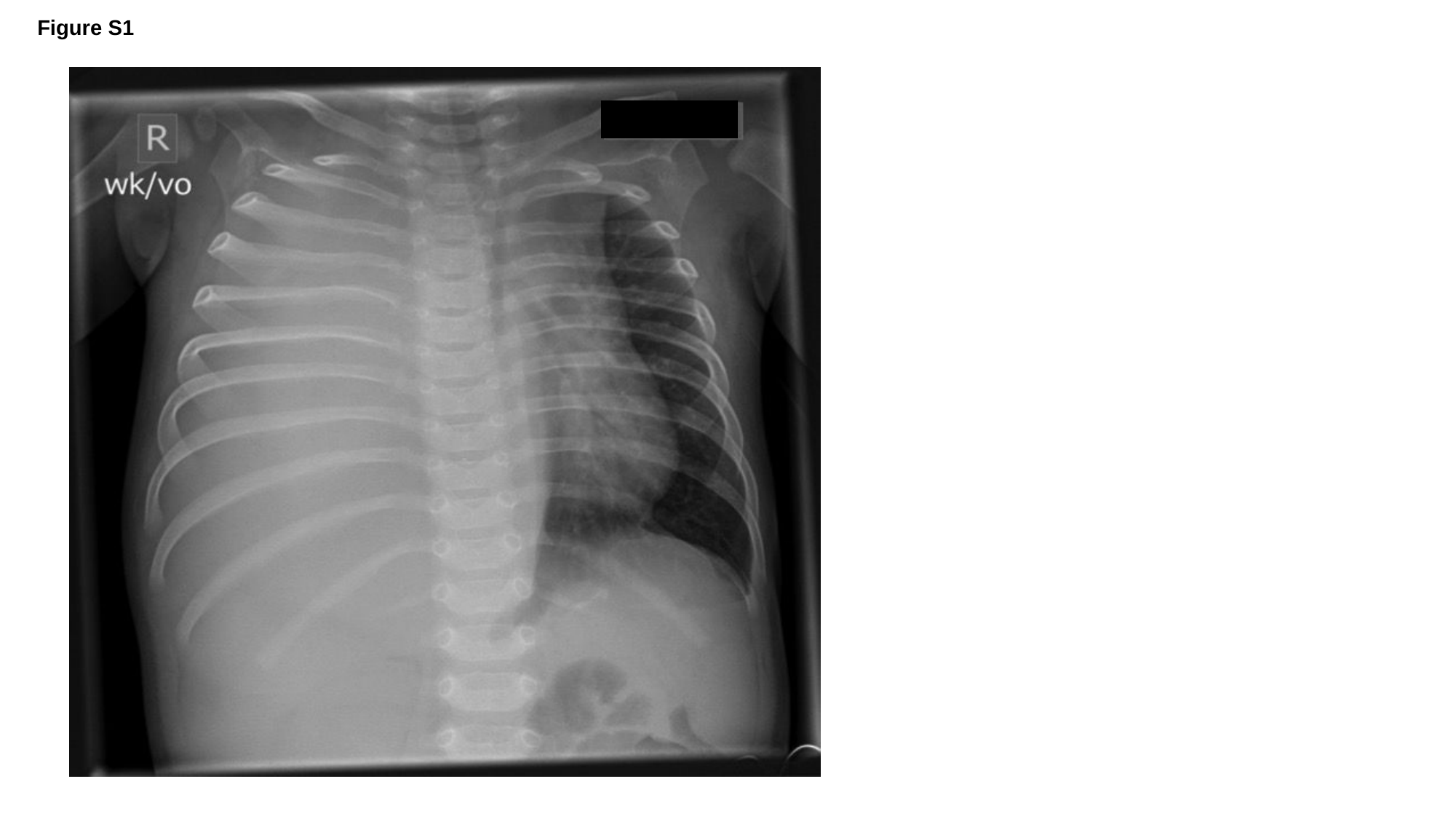

Figure S1

## Slide 2
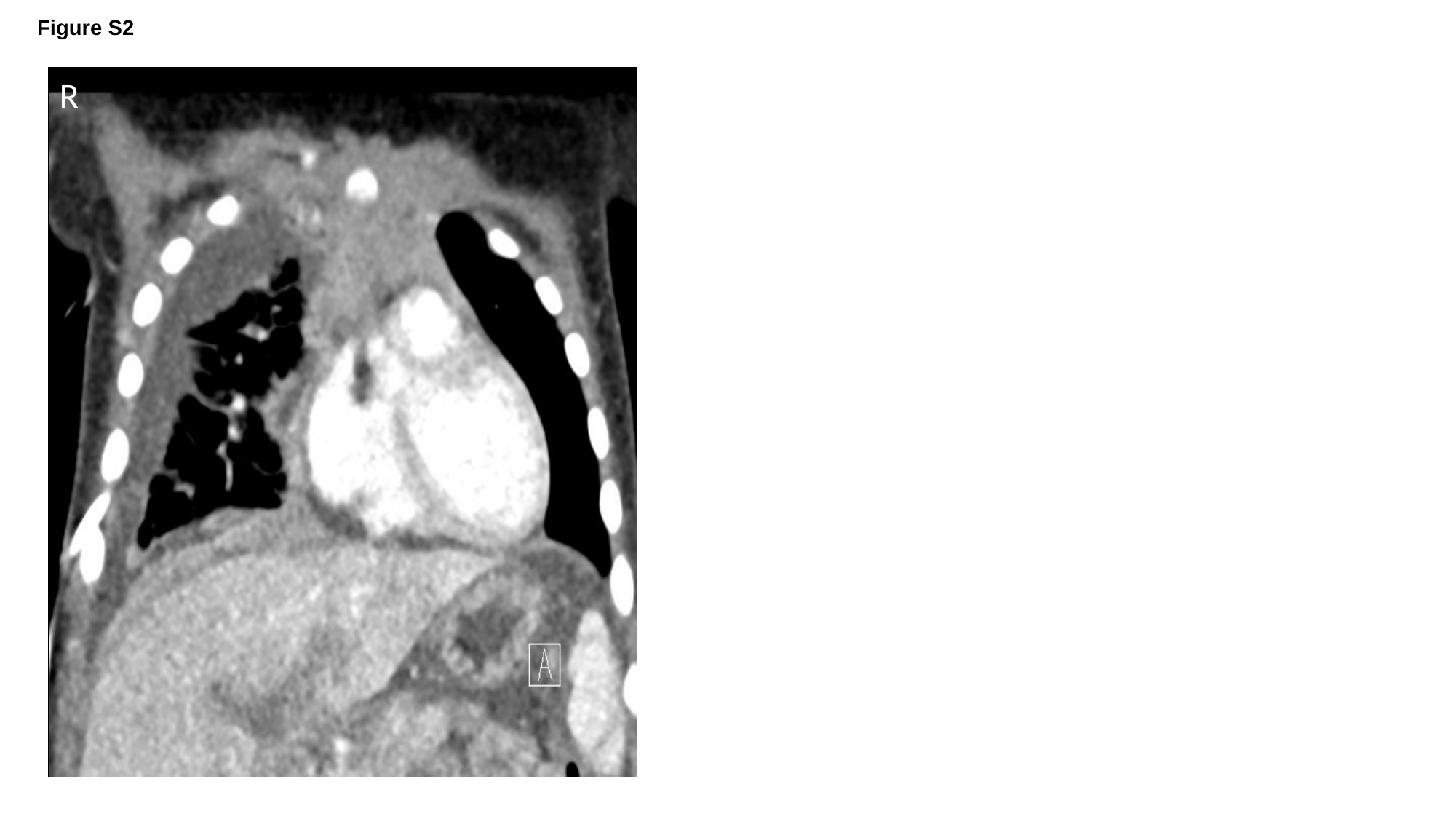

Figure S2
R

## Slide 3
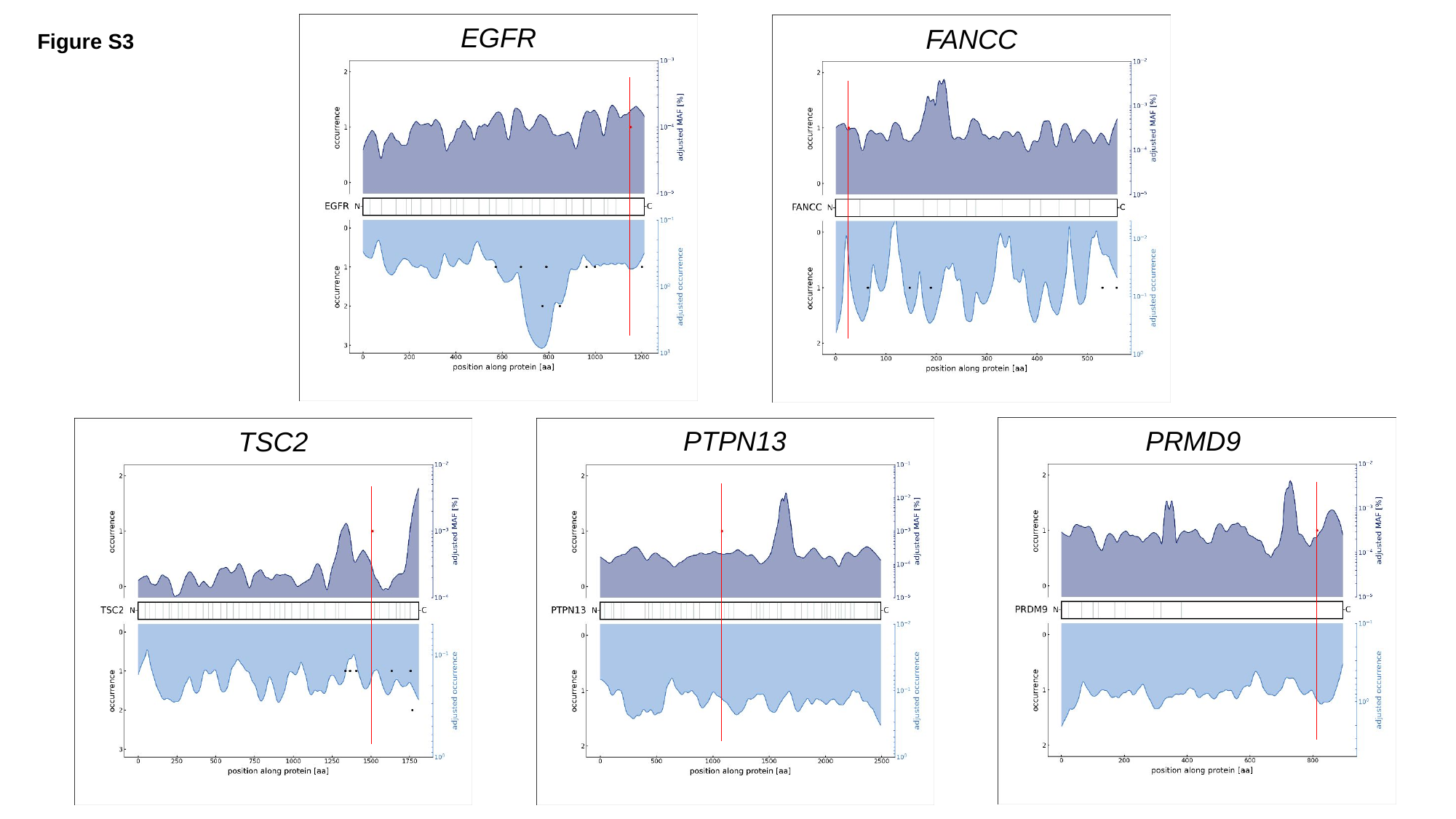

EGFR
FANCC
Figure S3
PRMD9
TSC2
PTPN13

## Slide 4
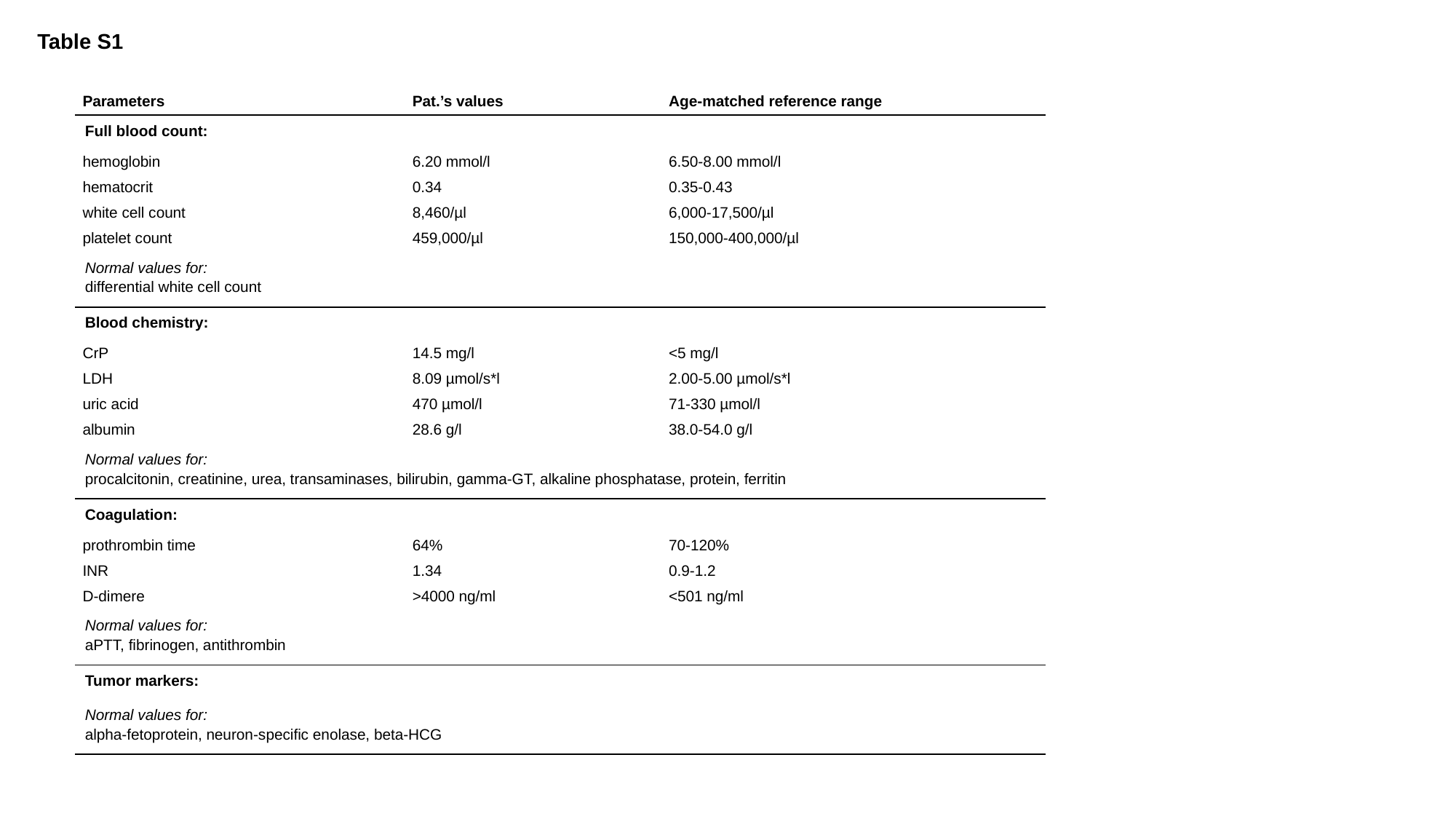

Table S1
| Parameters | Pat.’s values | Age-matched reference range |
| --- | --- | --- |
| Full blood count: | | |
| hemoglobin | 6.20 mmol/l | 6.50-8.00 mmol/l |
| hematocrit | 0.34 | 0.35-0.43 |
| white cell count | 8,460/µl | 6,000-17,500/µl |
| platelet count | 459,000/µl | 150,000-400,000/µl |
| Normal values for: differential white cell count | | |
| Blood chemistry: | | |
| CrP | 14.5 mg/l | <5 mg/l |
| LDH | 8.09 µmol/s\*l | 2.00-5.00 µmol/s\*l |
| uric acid | 470 µmol/l | 71-330 µmol/l |
| albumin | 28.6 g/l | 38.0-54.0 g/l |
| Normal values for: procalcitonin, creatinine, urea, transaminases, bilirubin, gamma-GT, alkaline phosphatase, protein, ferritin | | |
| Coagulation: | | |
| prothrombin time | 64% | 70-120% |
| INR | 1.34 | 0.9-1.2 |
| D-dimere | >4000 ng/ml | <501 ng/ml |
| Normal values for: aPTT, fibrinogen, antithrombin | | |
| Tumor markers: | | |
| Normal values for: alpha-fetoprotein, neuron-specific enolase, beta-HCG | | |

## Slide 5
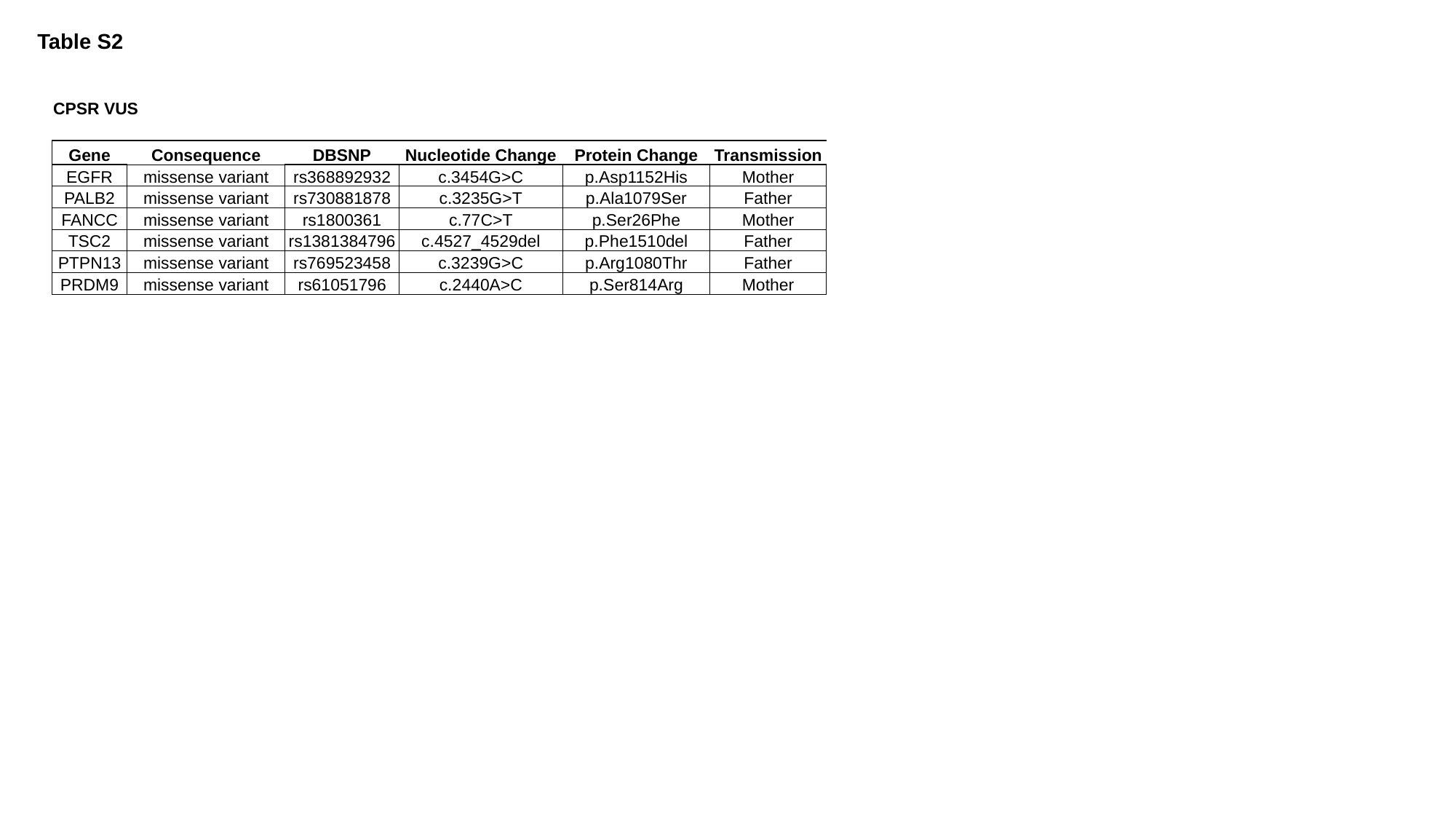

Table S2
| CPSR VUS | | | | | |
| --- | --- | --- | --- | --- | --- |
| | | | | | |
| Gene | Consequence | DBSNP | Nucleotide Change | Protein Change | Transmission |
| EGFR | missense variant | rs368892932 | c.3454G>C | p.Asp1152His | Mother |
| PALB2 | missense variant | rs730881878 | c.3235G>T | p.Ala1079Ser | Father |
| FANCC | missense variant | rs1800361 | c.77C>T | p.Ser26Phe | Mother |
| TSC2 | missense variant | rs1381384796 | c.4527\_4529del | p.Phe1510del | Father |
| PTPN13 | missense variant | rs769523458 | c.3239G>C | p.Arg1080Thr | Father |
| PRDM9 | missense variant | rs61051796 | c.2440A>C | p.Ser814Arg | Mother |
| | | | | | |
