# Supplementary material for: A rare PALB2 germline variant causing G2/M cell cycle arrest is associated with isolated myelosarcoma in infancy
Source: Mol Genet Genomic Med. 2021 Aug 12;9(9):e1746. doi: 10.1002/mgg3.1746 (PMC8457705; doi:10.1002/mgg3.1746)
Supplement: Supplementary file 2 — Supinfo S2 [file MGG3-9-e1746-s002.docx]

**Supplementary Information (*Beer et al*., 2021)**

**Material and Methods:**

**Ethical Compliance:**

Families were consented according to the Ethical Vote EK 181042019 and in line with the Declaration of Helsinki.

**Cell culture:**

TRIO-DD-005 and TRIO-DD_021 primary Fibroblasts were first cultivated in BIO-AMF™-2 Medium (Biological-Industries) up to passage 4. X107 (Control Fibroblasts), TRIO-DD_021 and TRIO-DD-005 fibroblasts were then grown in Dulbecco’s Modified Eagle Medium (GIBCO) with 20% fetal calf serum (GIBCO) and 1% Penicillin/Streptomycin (10000 units/ml- GIBCO) and 1% MEM Non-essential Amino Acids (GIBCO) up to a passage of 14.

**Whole exome sequencing (WES):**

DNA was extracted from patient’s fibroblasts using AllPrep DNA/RNA Mini Kit (Qiagen) and from PBMCs of the parents using the QIAamp DNA Blood Mini Kit (Qiagen). Sequenceable next-generation libraries for WES were generated with the SureSelect Human All Exon V7 kit (Agilent). The libraries were sequenced on a NovaSeq 6000 platform (Illumina) in paired-end mode (2x150bp) and with a final on target coverage of ≥100x. After generation of read files in fastq format using bcl2fastq v2.19.0, trimmomatic v0.33 was used to remove adapter and low-quality sequences (Bolger, Lohse, & Usadel, 2014). The alignment to the human reference genome GRCh37 was performed using BWA-MEM v0.7.12 (Li & Durbin, 2009) and Samtools v1.2 (Li et al., 2009). The tool Peddy 0.4.6 (Pedersen & Quinlan, 2017) performed gender and relatedness analyses to validate the correct sample assignment and the expected relationship of the patients’ data with the corresponding parents’ data. Single nucleotide variants (SNVs) and insertion/deletions (indels) were called using GATK v4.1.4.1 and VarScan2 v2.3.9 (Koboldt et al., 2012), applying the trio-mode.

Initial variant interpretation was carried out with the CPSR pipeline (Nakken S, 2019), which classified the variants as pathogenic, likely pathogenic, variant of unknown significance (VUS), likely benign or benign. Additional variant interpretation was manually performed (e.g by taking CADD scores into account (Kircher et al., 2014)) as well as by utilizing an extended cancer gene list.

**Sanger Sequencing Validation:**

*PALB2* p.A1079S was validated via PCR and subsequent Sanger Sequencing using the following primers (5’🡪3’): hPALB2_Ex12_F (ACTTCAGAGCCTATCGGTCAT) and hPALB2_Ex12_R (CCACGCTGAGAGTCGTCTTA).

**Variation analysis:**

Allele frequencies of all coding germline variants present in e.g. *PALB2* in a global, healthy population, taken from gnomAD non-cancer exome r.2.1.1 dataset, are summed up codon-wise. Somatic, coding variants reported for an adult cancer cohort derive from COSMIC, GRCh37 Release 91 and are similarly combined for each codon along e.g. *PALB2*. Both collected datasets are smoothed using the LOWESS algorithm (fraction: 0.06, iterations: 3) prior to plotting.

**Irradiation and Cell cycle analysis**

Fibroblasts were seeded in T25 cm^2^ bottles at a density of 1-2x10^5^ cells and cultured at 37 °C, 5% CO_2_. After 48-72 hours depending on cell density cells were irradiated with 6 Gy. 48 h later, apoptosis was analyzed by propidium iodide staining and flow cytometry adapted from Riccardi and Nicoletti (Riccardi & Nicoletti, 2006). In short: cells were trypsinized (GibcoTrypLE Express), washed with PBS ((Dulbecco’s Phosphate Buffered Saline),centrifuged at 200 g 5 min at RT and fixated with 70% ice-cold Ethanol/PBS for at least 20 min (up to 1 h) at -20°C. Cells were then centrifuged (all centrifugation steps at 400g, 4 °C) and washed with cold PBS. After centrifugation, cells were treated with 500 µl of DNA extraction buffer (Nicoletti) and 500 µl of cold PBS. After 5 minutes incubation on ice cells were again centrifuged and resuspended in staining solution (PBS, 100 µg propium iodide (BioLegend- 421301), 1 mg DNA-free-RNAse (Thermo Scientific EN0531) and 2 mM EDTA –Nicoletti. Cells were incubated in the dark for at least 30 min before FACS analysis.

**Mitomycin C treatment and cell cycle analysis**

Fibroblasts were seeded on 10 mm plates for cell culture or T25 cm^2^ bottles at a density of 1.5-3 x 10^5^ cells and cultured at 37°C, 5% CO_2_. After 48 h, cells were washed with PBS. Mitomycin C from Streptomyces caespitosus (Sigma M4287) diluted in PBS to a concentration of 0.1 mg/ml was applied and cells were incubated at room temperature for 5 min. The untreated control was kept in PBS only. After 5 min, cells were washed 3x with PBS and cultured with fresh media at 37°C, 5% CO_2_ for 24 h, before apoptosis was analyzed by propidium iodide staining and flow cytometry as stated for the irradiation assay above.

**Supplementary Figures**

**Figure S1:** Chest x-ray on admission with complete opacity of the right chest and mediastinal shift to the left

**Figure S2:** Initial chest CT, coronary axis: Highly suspicious for a pleural and mediastinal lymphoma with an extensive pleural effusion and a small pericardial effusion

**Figure S3:** Distribution of mutational frequencies along the remaining VUS identified by CPSR (**Table S2**). Upper graph: combined minor allele frequencies of all coding germline variants from the gnomAD non-cancer database represented as LOWESS fit. Lower graph: combined and smoothed (LOWESS) occurrences of somatic tumor mutations from the COSMIC database. Black dots represent somatic variants present in pediatric cancer taken from the St. Judes PeCan database. The location of the respective germline variants is indicated as red line.

**Table S1:** Laboratory results on admission

**Table S2:** List of Cancer Predisposition Sequencing Reporter (CPSR) Variants of uncertain significance (VUS).

**References**

Bolger, A. M., Lohse, M., & Usadel, B. (2014). Trimmomatic: a flexible trimmer for Illumina sequence data. *Bioinformatics, 30*(15), 2114-2120. doi:10.1093/bioinformatics/btu170

Kircher, M., Witten, D. M., Jain, P., O'Roak, B. J., Cooper, G. M., & Shendure, J. (2014). A general framework for estimating the relative pathogenicity of human genetic variants. *Nat Genet, 46*(3), 310-315. doi:10.1038/ng.2892

Koboldt, D. C., Zhang, Q., Larson, D. E., Shen, D., McLellan, M. D., Lin, L., . . . Wilson, R. K. (2012). VarScan 2: somatic mutation and copy number alteration discovery in cancer by exome sequencing. *Genome Res, 22*(3), 568-576. doi:10.1101/gr.129684.111

Li, H., & Durbin, R. (2009). Fast and accurate short read alignment with Burrows-Wheeler transform. *Bioinformatics, 25*(14), 1754-1760. doi:10.1093/bioinformatics/btp324

Li, H., Handsaker, B., Wysoker, A., Fennell, T., Ruan, J., Homer, N., . . . Genome Project Data Processing, S. (2009). The Sequence Alignment/Map format and SAMtools. *Bioinformatics, 25*(16), 2078-2079. doi:10.1093/bioinformatics/btp352

Nakken S, S. V., Hofmann O, Møller P, Myklebost O, Hovig E (2019). Cancer Predisposition Sequencing Reporter (CPSR): a flexible variant report engine for germline screening in cancer Bioinformatics. *Available at:* [*http://biorxiv.org/lookup/doi/10.1101/846089*](http://biorxiv.org/lookup/doi/10.1101/846089) *[Accessed July 29, 2020]*.

Pedersen, B. S., & Quinlan, A. R. (2017). Who's Who? Detecting and Resolving Sample Anomalies in Human DNA Sequencing Studies with Peddy. *Am J Hum Genet, 100*(3), 406-413. doi:10.1016/j.ajhg.2017.01.017

Riccardi, C., & Nicoletti, I. (2006). Analysis of apoptosis by propidium iodide staining and flow cytometry. *Nat Protoc, 1*(3), 1458-1461. doi:10.1038/nprot.2006.238
